# The Boredom-ADHD Nexus: A Narrative and Meta-Analytic Review of the Evidence

**DOI:** 10.1007/s10567-026-00563-9

**Published:** 2026-03-11

**Authors:** Peter Muris, Henry Otgaar, Franc Donkers

**Affiliations:** 1 Department of Clinical Psychological Science, Faculty of Psychology and Neuroscience, Maastricht University, P.O. Box 616, 6200 MD Maastricht, The Netherlands; 2 Stellenbosch University, Stellenbosch, South Africa; 3 Youz-Parnassia Group Maastricht, Maastricht, The Netherlands; 4 Catholic University of Leuven, Louvain, Belgium

**Keywords:** Attention**-**deficit/hyperactivity disorder (ADHD), Boredom, Meta-analysis, Narrative review

## Abstract

This paper examines the intersection of attention-deficit/hyperactivity disorder (ADHD) and boredom. We begin with a short overview of ADHD, describing its core symptoms, cognitive features, and biological underpinnings, followed by discussion of boredom as a psychological construct, with an emphasis on individual differences in boredom propensity. Next, we review existing empirical studies and present new meta-analytic findings concerning the link between ADHD and boredom. Across 18 mostly correlational studies (total *N* = 22,365), an overall effect of *r* = 0.40 (95% CI: 0.35–0.46) was observed, indicating a statistically significant positive association between boredom and ADHD. Building on these findings, we propose an integrated theoretical framework explaining why individuals with ADHD may be particularly susceptible to boredom and how this susceptibility may affect motivation, emotion regulation, and goal-directed behavior. Finally, we discuss clinical implications and describe key directions for future research. The current work highlights boredom as a central, rather than peripheral, experience in ADHD, with important implications for theory building, assessment practices, and intervention.

## Introduction

Boredom is an aversive cognitive-affective condition characterized by weariness, restlessness, and a yearning for meaningful engagement, arising when available activities fail to provide sufficient interest, stimulation, or value (Eastwood et al., 2012). Despite its ubiquity, boredom was long neglected as a topic of scientific inquiry (Westgate & Steidle, 2020). William James, whose work helped establish the empirical study of human emotion, largely dismissed boredom as a “real” emotion because it lacked the pronounced bodily changes associated with typical emotions such as fear, anger, or joy (James, 1894; 1994). In contrast, contemporary theories increasingly regard boredom as a legitimate emotional phenomenon that emerges when individuals appraise their situation as unstimulating or lacking meaning. Specifically, current theoretical models suggest that boredom engages reward, attentional, and affective neural circuits (Anderson & Perone, 2024; Eastwood et al., 2012; Milyavskaya et al., 2019; Westgate & Wilson, 2018) and serves a functional role by motivating disengagement from unsatisfying contexts in pursuit of more meaningful alternatives (Scarantino, 2024). This reconceptualization has stimulated increased empirical attention in boredom over recent decades, expanding research into its causes, correlates, and consequences (Danckert, 2013; Danckert & Eastwood, 2020).

One of the earliest descriptive studies of boredom was conducted by Harris (2000), who surveyed 170 college students about their everyday experiences with the phenomenon. Participants frequently reported the prototypical features of boredom—such as fatigue, unease, and wandering attention—often accompanied by feelings of frustration and sadness, and occurring on average once per day. Boredom was most common during predictable, unchallenging, repetitive, or monotonous activities (e.g., lectures and classes), during periods of solitude, or while waiting. Only 10% (*n* = 17) of respondents indicated that they were never bored, whereas others experienced boredom up to ten times per day, highlighting substantial individual variation in the propensity to become bored. Participants also described a range of strategies for coping with boredom, including reading, reflecting or daydreaming, socializing, watching television, engaging in physical activity, or trying something new. These responses support the view that boredom can function as a catalyst for behavioral change, underscoring its potential adaptive or functional value.

Although boredom is typically an innocuous and transient experience, it becomes clinically meaningful when it occurs on a frequent base or is experienced with particular intensity. High levels of boredom proneness have been associated with a wide range of psychological and behavioral difficulties, including heightened anxiety and depression (Goldberg et al., 2011; Sommers & Vodanovich, 2000), alexithymia (Eastwood et al., 2007), somatic complaints (Sommers & Vodanovich, 2000), aggression (Freund et al., 2021), and impulse control problems such as overeating (Moynihan et al., 2015), substance abuse (Weybright et al., 2015), problematic internet use (Tagliaferri et al., 2025), and gambling (Blaszczynski et al., 1990). These associations suggest that boredom proneness may reflect broader challenges with self-regulation, emotional awareness, or engagement with one’s environment. For some individuals, boredom is not merely dull or unpleasant but profoundly aversive. This is especially the case for people with attention-deficit/hyperactivity disorder (ADHD), who often describe boredom as “torture,” as “almost painful, like wearing an itchy coat but you can’t scratch,” or as “a profound inner restlessness” that creates an urgent need to escape the situation. Others report that boredom and silence can feel as though “the walls are closing in,” or recall childhood boredom as “so overwhelming and unbearable that it elicited tears” (examples from ADDitude ADHD Science & Strategies, n.d.). These firsthand accounts highlight that boredom in ADHD is not simply a mild dislike of unstimulating contexts but a deeply distressing affective state. Consistent with these accounts, challenges with sustaining attention, regulating arousal, and maintaining motivation may render individuals with ADHD particularly vulnerable to chronic or intolerable boredom.

Building on this background, the present paper focuses specifically on the intersection of ADHD and boredom. We begin by providing a brief overview of ADHD as a neurodevelopmental disorder, outlining its core symptoms, cognitive characteristics, and relevant biological underpinnings. We then offer a detailed examination of boredom, with particular attention to individual differences in boredom propensity. Next, we review the existing empirical literature directly examining the association between ADHD and boredom and present new meta-analytic findings that quantify the strength and consistency of this link. Given the clinical features of ADHD and first-hand accounts from affected individuals, we expected to document a positive association between ADHD and boredom. Drawing on theoretical accounts of boredom and neurobiological models of ADHD, we then propose an integrated framework that explains how the processes underlying both constructs may interact. Finally, we discuss the implications of this work for clinical practice and identify key directions for future research aimed at advancing our understanding of boredom in the context of ADHD.

## ADHD: Disorder or Natural Variation?

ADHD is a neurodevelopmental disorder defined by persistent patterns of inattention and/or hyperactivity-impulsivity (American Psychiatric Association [APA], 2022). Inattention may involve difficulty sustaining focus, becoming easily distracted, forgetting daily responsibilities such as appointments or schoolwork, struggling to organize activities or manage time, or frequently losing important items like keys, books, or assignments. Hyperactivity-impulsivity may manifest as restlessness, difficulty remaining seated, excessive talking, a constant sense of being “on the go”, interrupting others, difficulty waiting one’s turn, or acting without considering consequences. ADHD has a childhood onset, with symptoms emerging before age 12. These difficulties must be evident in multiple settings—such as school, work, at home, or social environments—and must persist across development (APA, 2022). Although overt hyperactive behavior often diminishes during adolescence and adulthood, problems with inattention, restlessness, and impulsivity frequently continue (Mick et al., 2004). To meet diagnostic criteria, the symptoms must also cause clinically significant impairment, such as academic underachievement, workplace difficulties, strained relationships, or chronic problems with organization and time management (APA, 2022).

ADHD is a common mental health condition. A recent umbrella review of meta-analyses of 588 studies including more than three million participants estimated the global prevalence of ADHD in children and adolescents at 8%, although estimates varied considerably across studies (3.4%–14%). Prevalence was approximately twice as high in boys (10%) as in girls (5%; Ayano et al., 2023a). A parallel umbrella review of research conducted in adults, synthesizing 57 unique studies and over 21 million participants, reported a somewhat lower pooled prevalence of 3.1% (ranging from 2.2%–5%; Ayano et al., 2023b). Together, these findings indicate that while ADHD symptoms often decline with age, the condition remains a substantial concern across the lifespan.

Historically, theories of ADHD have been strongly biologically oriented, proposing that its prototypical symptoms stem from atypicalities in brain structure and function. Shaw and Szekely (2018), for example, summarized evidence suggesting that ADHD is associated with smaller-than-average volumes of the prefrontal cortex, striatum, and cerebellum—regions implicated in inhibition, working memory, planning, delay aversion, and temporal processing, all domains in which individuals with ADHD commonly experience difficulty. At a functional level, ADHD has also been linked to dysregulation of dopamine and noradrenaline, neurotransmitters central to brain circuits supporting executive functions, reward processing, and motivation. Inefficient signaling in these systems has been associated with impaired executive control, reduced sustained attention, altered reward sensitivity, and diminished task-related motivation (Prince, 2008). Given this longstanding biological orientation, ADHD has traditionally been conceptualized as a neurodevelopmental disorder that warrants medical evaluation and intervention (Mahone & Denckla, 2017).

In recent years, however, the neurodivergent perspective on neurodevelopmental conditions such as ADHD has gained increasing traction (Fletcher-Watson, 2022; Morris et al., 2025a, 2025b). This framework does not deny the difficulties ADHD can cause but emphasizes that ADHD may represent a natural variation in cognitive functioning rather than a disorder in the traditional sense. In this view, context plays a central role in determining whether ADHD-related traits become impairing or, alternatively, can be expressed as strengths. Environments requiring prolonged sedentary work, rigid attention, or strict compliance may amplify challenges, whereas settings that value creativity, rapid problem-solving, novelty-seeking, or deep engagement with personally meaningful tasks may allow the same traits to become advantageous. Thus, the neurodivergent framework shifts the focus from individual deficits to a misalignment between individual cognitive styles and environmental expectations (Armstrong, 2010; Ellis, 2023; Nerenberg, 2021).

This perspective offers a useful explanation for why individuals with high ADHD traits are particularly prone to boredom in conventional school and work contexts. Such environments often lack novelty, autonomy, meaningful stimulation, or opportunities for self-directed engagement—conditions under which individuals high on the ADHD spectrum tend to struggle. In these settings, boredom may arise as a persistent signal of under-stimulation and person-environment misfit, reflecting a discrepancy between the individual’s need for dynamic, interest-driven tasks and the monotonous and externally structure nature of many educational and occupational environments (Ellis et al., 2023; Patton & Santuzzi, 2024).

## Boredom and Boredom Propensity

Boredom is a negative affective experience characterized by a constellation of behavioral, cognitive, and emotional components. Behaviorally, it manifests as restlessness and/or disengagement, including actions such as fidgeting, shifting posture, looking around, or yawning. Cognitively, boredom is marked by difficulty sustaining attention, increased mind-wandering, reduced sense of task meaning, and the subjective impression that time is passing slowly. Emotionally, boredom involves irritation, apathy, and dissatisfaction, reflecting a motivational state in which individuals desire greater stimulation or engagement but lack a clear direction for achieving it (Danckert, 2013; Danckert & Eastwood, 2020). There are marked individual differences in the propensity to experience boredom (Hunter et al., 2016). Boredom propensity is distinct from state boredom, which is the transient experience of boredom in a specific moment (Fahlman et al., 2013), whereas boredom propensity reflects a more generalized tendency or disposition toward experiencing boredom (Tam et al., 2021).

The most widely used instrument for assessing boredom propensity is the *Boredom Proneness Scale* (BPS; Farmer & Sundberg, 1986), a 28-item measure designed to capture the general tendency to feel bored across diverse contexts. Although the BPS has demonstrated solid reliability and validity, several issues with the scale have been noted. Most notably, its structure appears complex and does not reflect a single underlying factor but instead comprises multiple components. While some studies have identified as many as eight factors (see Melton & Schulenberg, 2009), a two-factor solution has been most consistently reported (Vodanovic, 2003; Vodanovic & Watt, 2016). These two factors are *lack of internal stimulation*, referring to an individual’s difficulty in self-generating interest and engagement, and *lack of external stimulation*, describing the inability to satisfy a strong need for excitement, challenge, and change (Vodanovic & Kass, 1990). The former boredom type has also been described as *apathetic* boredom, a subdued, low-arousal state marked by disengagement and diminished motivation, whereas the latter has been characterized as *agitated* boredom, a high-arousal state associated with restlessness, frustration, and a persistent drive to seek novelty or stimulation (Danckert, 2013).

In response to the psychometric inconsistencies of the original BPS, Struk et al. (2017) argued that part of the psychometric complexity stemmed from the inclusion of reverse-scored items, which can introduce methodological artefacts and obscure the true factor structure. To address this issue, they developed an eight-item Short Form of the BPS (BPS-SF) by removing all reverse-scored items as well as several items considered redundant or insufficiently aligned with the theoretical construct of boredom proneness. The resulting scale demonstrated clear one-dimensionality and strong psychometric properties, providing a concise yet robust measure of boredom propensity. Importantly, none of the BPS-SF items reference attention-related difficulties, making the scale particularly advantageous in research examining associations with ADHD, as it reduces the risk of measurement confounding arising from overlapping item content.

Another frequently used scale for assessing boredom propensity is the *Boredom Susceptibility Scale* (BSS), a subscale of Zuckerman’s (1979) Sensation Seeking Scale. The BSS measures an individual’s inability to tolerate monotonous environmental stimulation and thus represents only a narrower facet of the broader boredom propensity construct. It is similar to the BPS factor reflecting a ‘lack of external stimulation’, but it does not capture a ‘lack of internal stimulation’. Consequently, the correlation between the BPS and BSS is positive but typically modest, and external validity evidence indicates that the two scales do not assess the same construct. While the BPS appears more strongly associated with emotionality, avoidance, and attentional difficulties, BSS is more closely linked to reward sensitivity, motor impulsivity, and risk taking (Mercer-Lynn et al., 2013).

## Meta-Analytic Review of the Association Between Boredom and ADHD

**Fig. 1:**
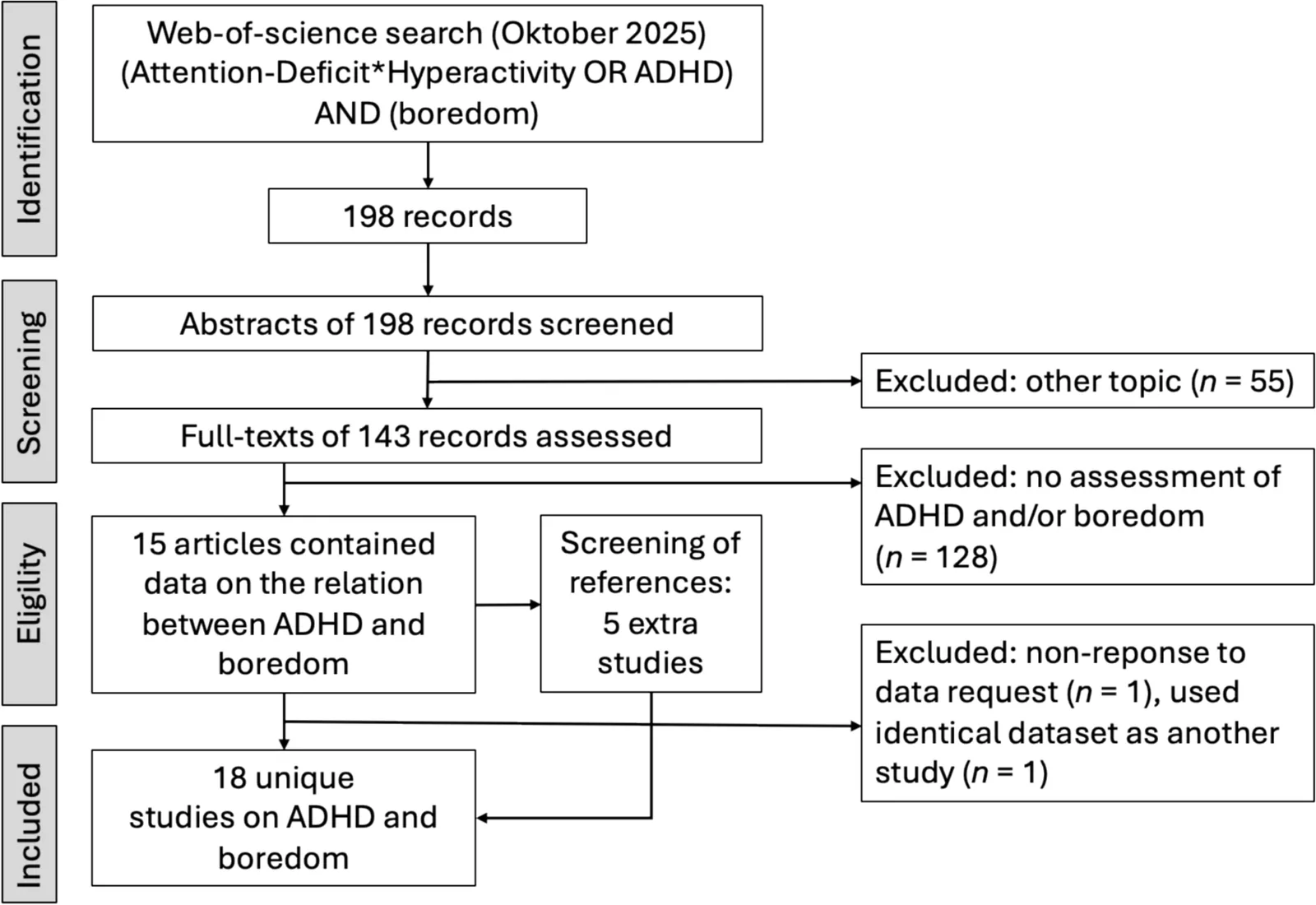
PRISMA flow diagram depicting the selection of articles on the relation between boredom and ADHD. *Note*. ADHD = Attention-deficit/hyperactivity disorder

The anecdotical accounts described earlier suggest that individuals with ADHD experience boredom more frequently and more intensely than those with the disorder. As Diamond’s (2005) noted, people suffering from this neurodevelopmental disorder “are easily bored” (p. 819; see also Danckert & Eastwood, 2020). To obtain an empirical picture of the relationship between boredom and ADHD, a literature search was conducted in Web-of-Science in November 2025 using the search terms “boredom” (Topic] AND “attention-deficit*” OR “ADHD” (Topic). As shown in the PRISMA flow diagram (Page et al. 2021; Fig. 1), this search yielded 198 records. Screening of these records identified 15 studies that assessed both boredom and ADHD. The scope of our search was intentionally broad: we included not only research on boredom propensity but also studies assessing state boredom or boredom in specific contexts (e.g., work, sex, gaming, education). Two studies were excluded—one because the authors did not provide the requested correlation between boredom proneness and ADHD symptoms (Moon et al., 2017), and another because it used the same dataset as a study already included (Gobulchik et al., 2021). Examination of the reference lists of retrieved studies yielded five additional articles with relevant data, resulting in a final total of 18 studies.

The included studies comprised a total of *N* = 22,365 participants, with an overview presented in Table 1. As shown, most studies focused on adult samples (*k* = 14), whereas only four examined children and adolescents. Notably, the majority of studies included more female than male participants (*k* = 11), which contrasts the typically higher prevalence of ADHD and related traits among males (Babinski, 2024). Most samples were typically developing individuals (*k* = 14), particularly students (*n* = 9), and only three studies involved clinically referred participants. Regarding design, the evidence on the relation between boredom and ADHD were predominantly correlational (*k* = 15), with only three studies directly comparing boredom levels between ADHD and control groups. Boredom was most commonly conceptualized as boredom propensity (*k* = 14), typically assessed using the BPS (either the original or Short Form; *k* = 13). In contrast, state boredom (*k* = 3) and context-specific boredom (*k* = 4) were less frequently measured. Finally, assessments relied chiefly on self-reports (*n* = 14), with only four studies incorporating reports from other informants (in all cases, parents).

**Table 1:** Summary of empirical studies on the relationship between boredom and ADHD

Study	Sample	Method	Boredom type	Boredom assessment	ADHD assessment
Chan and Langberg (2024)	100 adults with ADHD (49 females, 51 males), 19–30 years (*M* = 26.6)	Online, correlational	Work-related boredom	Dutch Boredom Scale	Barkley Adult ADHD Rating Scale-IV
Chou et al. (2018)	300 adolescents with ADHD (41 girls, 259 boys), 10–18 years (*M* = 12.8)	Clinic, correlational	Boredom propensity	Boredom Proneness Scale-Short Form	Swanson Nolan and Pelman (SNAP-IV) Rating Scale*
De Oliveira et al. (2025)	1293 adults (722 females, 564 males), 18–94 years (*M* = 47.3)	Online, correlational	Sexual boredom	Sexual Boredom Inventory	Adult ADHD Self-Report Scale
Fahlman et al. (2013)	243 adults (134 females, 109 males), 17–56 years (*M* = 20.0)	Student, correlational	State boredom, boredom propensity	Multidimensional State Boredom Scale, Boredom Proneness Scale	Adult ADHD Self-Report Scale
Gerritsen et al. (2014)	131 adults (97 females, 34 males), 17–45 years (*M* = 21.2)	Student, correlational	Boredom propensity	Boredom Susceptibility Scale, Boredom Proneness Scale, Multidimensional Trait Boredom Scale	Adult ADHD Self-Report Scale
Golubchik et al. (2020)	30 children and adolescents with ADHD (9 girls, 21 boys), 7–18 years (*M* = 12.2)	Clinic, correlational, treatment	Boredom propensity	Boredom Proneness Scale-Short Form	ADHD Rating Scale for DSM-5*
Hsu et al. (2025)	93 children and adolescents with ADHD and 90 typically developing controls (28 girls, 155 boys), 9–16 years (*M* = 12.1)	Clinic and community, group comparison	Boredom propensity	Boredom Proneness Scale-Short Form**	ADHD versus control
Kass et al. (2003)	148 adults (78 females, 70 males), *M* age = 22.7 years	Student, correlational	Boredom propensity	Boredom Proneness Scale	Adult Behavior Checklist
Koncz et al. (2024)	14,740 adults (1581 females, 13,157 males), *M* age = 24.1 years	Online, correlational	Boredom gaming motivation	Gaming Motivation Inventory	Adult ADHD Self-Report Scale
Malkovsky et al. (2012)	48 adults (39 females, 9 males), *M* age = 18.9 years	Student, correlational	Boredom propensity	Boredom Proneness Scale	Conners’ Adult ADHD Rating Scale
Müller et al. (2023)	215 adults with ADHD and 193 controls (274 females, 134 males), *M* age = 23.4 years	Student, group comparison	Academic boredom	Short College Boredom Scale	ADHD versus control
Orban et al. (2025)	88 adults (70 females, 18 males), *M* age = 18.9 years	Student, correlational	Boredom propensity	Boredom Proneness Scale-Short Form	Barkley Adult ADHD Rating Scale-IV, Adult ADHD Self-Report Scale, Wender Utah Rating Scale
Seiler et al. (2025)	19 adults with ADHD and 141 healthy controls (124 females, 36 males), *M* ages 32.7 and 22.7	Clinic and student, group comparison	State boredom, boredom propensity	Multidimensional State Boredom Scale, Boredom Proneness Scale	ADHD versus control
Struk et al. (2017)	2440 adults (1772 females, 668 males), *M* age = 20.2 years	Student, correlational	Boredom propensity	Boredom Proneness Scale-Short Form	Adult ADHD Self-Report Screener
Uehara and Ikegaya (2025)	341 children (169 girls and 172 boys), *M* age = 8.3 years	Community, correlational	Boredom propensity	Single-item measure of boredom tendency*	ADHD Rating Scale for DSM-IV*
Wallace et al. (2002)	335 adults (167 females, 168 males), *M* age = 22.2 years	Student/army personnel, correlational	Boredom propensity	Boredom Proneness Scale	Adult Behavior Checklist
Xu et al. (2025)	563 adults (280 females, 280 males), 18–26 years (*M* = 19.9)	Student, correlational	Boredom propensity	Boredom Proneness Scale-Short Form	Adult ADHD Self-Report Scale
Zerr et al. (2024)	814 adults (537 females, 277 males), *M* age = 41.5 years	Community, correlational	State boredom, boredom propensity	Multidimensional State Boredom Scale, Boredom Proneness Scale	Conners’ Adult ADHD Rating Scale

*: parent rating, **: parent and child/adolescent self-report. All other assessments relied solely on self-report

**Fig. 2:**
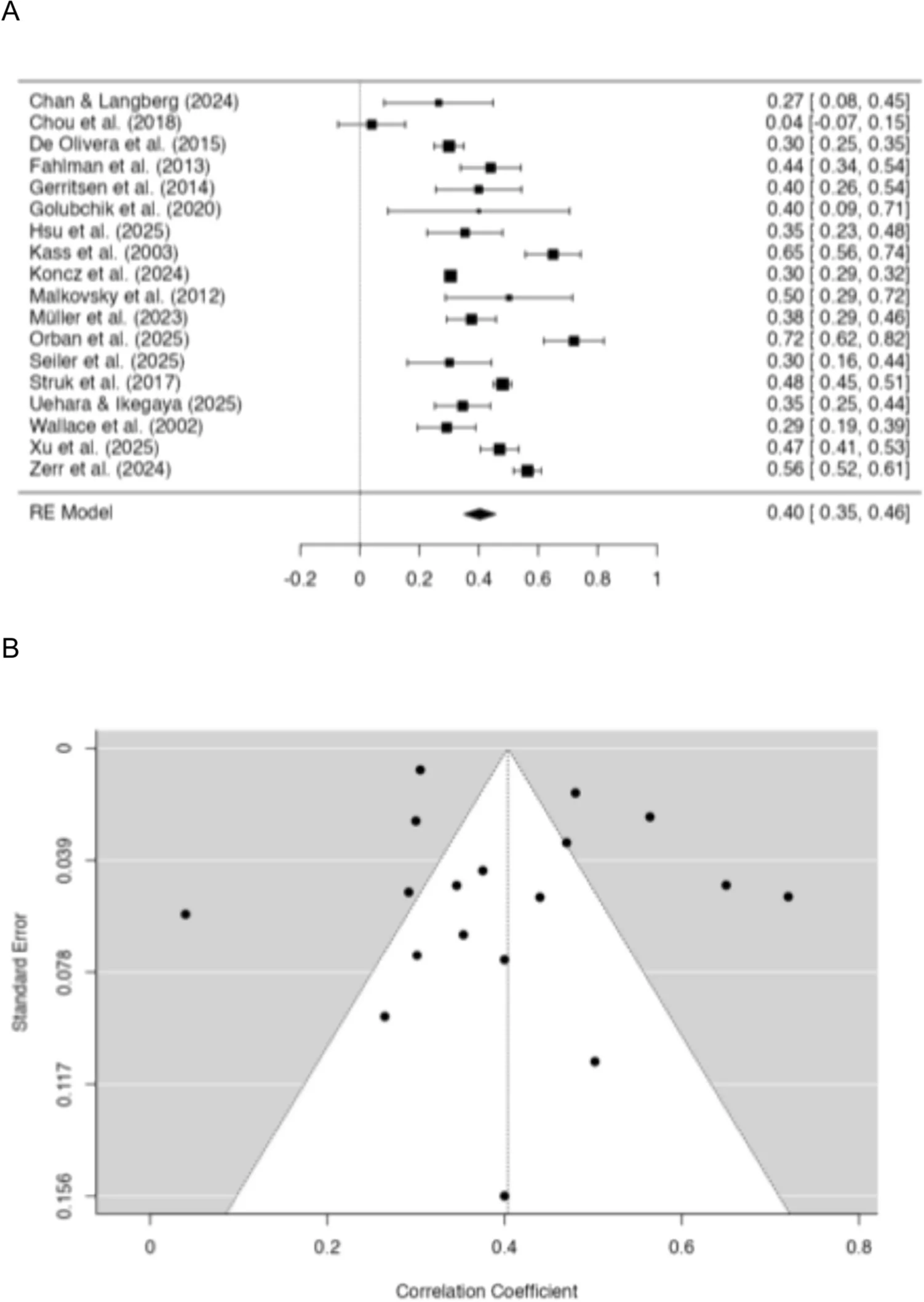
Forest plot (Panel **A**) and funnel plot (Panel **B**) of the meta-analysis on the relation between boredom and ADHD

Wilson’s (2023) online meta-analysis effect size calculator was used to compute the Fisher’s *z*-transformed correlation coefficients (*r*) and their corresponding 95% confidence intervals (CIs) for the association between boredom and ADHD reported in each study. In cases where a study provided multiple effect sizes for this relationship, the *r* values were first averaged before being entered into the calculator. Panel A of Fig. 2 presents the forest plot summarizing the effect sizes across the included studies. The meta-analysis was performed using Jamovi (The Jamovi Project, 2025) A random-effects model (Hunter-Schmidt) revealed a significant aggregate effect size of *r* = .40 was obtained, with a 95% CI of 0.35 to 0.46, indicating a significant positive association between boredom and ADHD (*Z* = 14.73, *p* < .001). Using the Binomial Effect Size Display (Rosenthal & Rubin, 1982), the aggregate effect was translated into a probability difference. An effect size of *r* = .40 suggests that approximately 70 out of 100 individuals with ADHD or elevated ADHD symptoms would be expected to fall in the high-boredom range, compared to about 30 out of 100 individuals without ADHD or with low ADHD symptom levels. This corresponds to a moderately strong association between ADHD and boredom. However, considerable heterogeneity was present across studies (Q = 495.45, *p* < .001; I^2^ = 93.87%), suggesting substantial variability in effect sizes. However, all individual study effects were directionally consistent with the positive pooled estimate. Neither the rank correlation test nor the regression test indicated funnel plot asymmetry (Fig. 2, Panel B). Nevertheless, Cook distances suggested that two studies (Chou et al., 2019; Orban et al., 2025) may have exerted disproportionate influence on the overall results. Notably, these studies exerted opposing influences on the pooled effect size: the study by Chou et al. tended to decrease the overall estimate, whereas the study by Orban et al. tended to increase it.

Taken together, these findings provide robust evidence for a meaningful and consistently positive link between boredom and ADHD, even though the magnitude of this association varies across studies. To better understand the sources of this variation, the next section examines the included studies in greater detail. This closer inspection highlights differences in study characteristics and methodological approaches, helping to clarify the diversity of observed effects.

## A Narrative Look at Boredom and ADHD

### Clinical Studies

Although ADHD is increasingly conceptualized as a dimensional phenomenon (Arildskov et al., 2024; Marcus & Barry, 2011) and can therefore be studied in typically developing populations, researchers should still prioritize examining its clinical manifestations. Our literature review indicates that only four studies investigating the relationship between boredom and ADHD have included clinically referred cases. Three of these focused on children and adolescents with the disorder. Of particular interest is the study by Golubchik et al. (2020), which assessed a small sample of children and adolescents with ADHD (*N* = 30, ages 7–18) using parent-reported ADHD Rating Scale for DSM-5 scores (DuPaul et al., 2016) and self-reported BPS-SF scores at three time points: baseline, after three months of methylphenidate treatment, and post-treatment discontinuation. The study highlighted a strong link between boredom and ADHD. At baseline, children’s self-reported boredom proneness was significantly positively correlated with parent-reported ADHD symptom severity. Both boredom proneness and ADHD severity decreased significantly during the methylphenidate treatment, with reductions in boredom closely parallelling reduction in ADHD symptoms. Finally, discontinuing the medication led to a resurgence of both boredom and ADHD symptom severity, further underscoring the intimate connection between these constructs.

In another study, Chou et al. (2018) examined 300 clinically referred adolescents with ADHD (aged 11–18 years), who completed the BPS-SF while their parents filled out the Swanson, Nolan, and Pelham (1981) Rating Scale-IV as an index of their child’s ADHD symptom severity. This was the only study that did not find a significant correlation between boredom proneness and ADHD. An important caveat, however, is that the assessments were conducted after the adolescents were already well into their clinical trajectory, with the vast majority (*n* = 254, 84.7%) receiving ADHD medication. As a result, symptoms were likely substantially reduced for many participants, which may have obscured the relationship between boredom proneness and ADHD. Another factor that may have contributed is the use of cross-informant data—namely, adolescents’ self-reported boredom paired with parent-rated ADHD symptoms—a method known to attenuate correlations between constructs (De Los Reyes et al., 2016).

Two recent clinical investigations have examined ADHD-control differences in boredom by comparing groupwise boredom scores. Hsu et al. (2025) assessed parent- and self-report BPS-SF scores in 93 children and adolescents with ADHD and 90 typically developing controls (aged 9–16 years). Seiler et al. (2025) compared an adult ADHD outpatient cohort (*n* = 19) with a healthy control student cohort (*n* = 141) on both boredom proneness (full-length BPS) and state boredom (Multidimensional State Boredom Scale; Fahlman et al., 2013). Hsu et al. (2025) found that young people with ADHD exhibited significantly higher boredom proneness scores than controls, with a notably stronger effect for parent-reported than for self-reported boredom (*Z* = 3.69, *p* < .001). Similarly, Seiler et al. (2025) reported elevated boredom scores among adults with ADHD relative to control participants, with effect sizes for boredom proneness and state boredom being comparable in magnitude (*Z* = .96, *p* = .17).

## Non-clinical Studies

Most research on the relationship between boredom and ADHD has been conducted with non-clinical participants, typically students or children and adults recruited online or through advertisements for experimental studies. For instance, during the psychometric evaluation of the BPS-SF, Struk et al. (2017) examined boredom proneness and ADHD symptoms using a brief six-item ADHD screener (Kessler et al., 2005) in a large sample of 2,440 undergraduate students. They found a positive correlation between boredom proneness and ADHD symptomatology (*r* = .48). A more recent study by Orban et al. (2025), also based on a student sample, used three ADHD rating instruments—the same ADHD screener, the Barkley Adult ADHD Rating Scale (Barkley, 2011), and the Wender Utah Rating Scale (Ward, 1993)—to classify participants in high- and low-ADHD-trait groups. In addition to these measures, participants completed the BPS-SF as an index of boredom propensity, along with several performance-based cognitive tasks assessing attention control and working memory. Their results showed a strong association between BPS-SF scores and the (combined) ADHD symptom measures (*r* = .72). However, this correlation was likely inflated due to the extreme-groups selection process (i.e., high- vs. low-ADHD-trait groups). Finally, Uehara et al. (2025) examined the association in a developmental context by collecting maternal reports on their children (mean age = 8.3 years). They assessed children’s boredom tendency using a single-item measure and ADHD symptoms with the DSM-IV-based ADHD Rating Scale (DuPaul et al., 2016). Once again, the findings indicated a positive relationship (*r* = .35): the more ADHD symptoms mothers reported, the more frequently they judged their child to experience boredom in daily life.

Whereas the studies described above focused on the relationship between boredom propensity and overall ADHD symptom levels, several investigations have examined how boredom relates specifically to the two core symptom clusters—inattention and hyperactivity-impulsivity. In an early study, Kass et al. (2003) administered the full-length BPS together with the Adult Behavior Checklist—a self-report measure of DSM-IV-defined ADHD symptoms (Barkley, 1998) in a sample of 148 university students. Inattention and hyperactivity-impulsivity symptoms were similarly associated with overall boredom proneness, with correlations of .63 and .59, respectively. These findings suggest that both symptom clusters are linked to an increased propensity for boredom (see also Gerritsen et al., 2014; Malkovsky et al., 2012; Wallace et al., 2002; Xu et al., 2025). A more nuanced picture emerged when the authors examined the BPS subscales *lack of internal stimulation* and *lack of external stimulation*. Inattention symptoms showed a stronger association with lack of internal stimulation, whereas hyperactivity-impulsivity symptoms were more substantially related to lack of external stimulation—a finding likewise reported by Malkovsky et al. (2012). This pattern implies that individuals with higher levels of inattention may struggle to generate or sustain internal engagement, whereas those with elevated hyperactivity-impulsivity symptoms may be more dependent on externally stimulating environments to maintain interest.

Given that boredom propensity reflects a general tendency to experience this negative emotion across a wide range of situations—and that individuals with ADHD score higher on this trait—one would expect ADHD to be accompanied by elevated boredom in specific contexts as well. We have already noted that ADHD has been linked to higher levels of state boredom (Fahlman et al., 2013; Zerr et al., 2024), and several studies have examined how this relationship unfolds in particular domains. For example, Chan and Langberg (2024) found that in adults with ADHD who were employed full time (*N* = 100) higher levels of hyperactivity/impulsivity were associated with greater boredom at work. In the context of gaming and smartphone addition, higher ADHD symptom levels have also been associated with more intense academic boredom (Müller et al., 2023) and a stronger tendency to use electronic devices for recreational—as well as procrastinatory—purposes (Koncz et al., 2024; Müller et al., 2023). Finally, Oliveira et al. (2025) recruited a large sample of U.S. residents (*N* = 1293) who completed a self-developed Sexual Boredom Index and the extended version of Adult ADHD Self-Report scale (Kessler et al., 2005). Their results showed a modest but significant positive correlation between sexual boredom and ADHD symptoms (*r* = .30), suggesting that individuals with higher symptom levels perceive sex as more monotonous and less exciting.

Altogether, the narrative review shows that research on the relationship between boredom and ADHD has been conducted in both clinical and non-clinical samples, at an approximate ratio of 1:5. Studies explicitly examining the role of boredom in ADHD remain a minority (*k* = 8, 44%); all four clinical studies fell into this category (100%), whereas in non-clinical studies, the link between boredom and ADHD symptoms was mostly a by-product of larger research projects (*k* = 10, 71%). Despite the relatively limited number of studies, a consistent positive link has been found between ADHD and boredom, applying not only to boredom propensity but also to state boredom and context-specific forms of boredom. Although the first study on this topic dates to 2002, overall scientific interest has remained modest, though a notable increase is evident in recent years, with 56% of studies (*k* = 10) conducted within the last three years. Building on this evidence of a robust association, the next section explores *why* boredom and ADHD are positively linked by reviewing theoretical explanations that may account for this relationship.

## Theoretical Perspectives on the Link Between Boredom and ADHD

This section postulates key theoretical perspectives on the relationship between boredom and ADHD, focusing on biologically rooted mechanisms as well as the experiential consequences that shape everyday functioning. These viewpoints are partly related yet also complementary, offering distinct angles on how boredom and ADHD intersect to heighten negative emotion and disruptive behavior. The section concludes with an integrated model illustrating how interacting processes underlying both constructs may converge to maintain this disorder and to magnify comorbid psychopathology.

## Cortical Hypoarousal

The cortical hypoarousal theory of ADHD proposes that many symptoms of the disorder stem from reduced baseline activity in key regions of the brain, particularly the prefrontal cortex. This reduced activation makes it more difficult for individuals to maintain alertness, sustain attention, and regulate impulses. Operating at a lower level of arousal than is optimal, people with ADHD may engage in compensatory behaviors—such as increased movement, fidgeting, or rapid shifts in focus—to elevate their arousal toward a more functional level (Satterfield et al., 1974). Supporting this model, a widely cited meta-analytic review of task-based functional Magnetic Resonance Imaging (fMRI) studies by Cortese et al. (2012) found consistent evidence that children and adults with ADHD show reduced activation in the frontoparietal regions relative to typically developing controls. Converging findings have emerged from electroencephalography (EEG) studies: hypoarousal is reflected in slower cortical responding and decreased event-related potential amplitudes (Mayer et al., 2016).

This chronic state of hypoarousal may help explain why people with ADHD appear to experience boredom more intensely and more quickly than others. It has been proposed that ADHD involves an underaroused baseline that makes sustained engagement difficult, particularly in low-stimulation environments. From this perspective, boredom might function as an experiential marker of hypoarousal: when tasks are routine, predictable, or insufficiently stimulating, they may fail to raise cortical activity to a comfortable or functional level. The resulting misalignment between task demands and arousal state could contribute to feelings of restlessness, dissatisfaction, and a drive to seek more engaging alternatives. In this view, boredom in ADHD is not merely a lack of interest, but potentially a neurophysiological signal that the brain is operating below an optimal arousal threshold, prompting compensatory attempts to increase stimulation and cognitive readiness (Danckert et al., 2018).

## Dominant Default Network Mode

Another account is concerned with the default mode network (DMN), which refers to a set of interconnected brain regions—involving the medial prefrontal cortex, posterior cingulate cortex and precuneus, inferior parietal lobule, and parts of the hippocampal system (Menon et al., 2023)—that are most active when an individual is not engaged in a specific, goal-directed task (Raichle et al., 2001). The term “default” reflects the idea that this network represents the brain’s *baseline mode of operation* when no particular task demands attention. Rather than being idle, the brain in this state is involved in internally oriented processes such as mind-wandering, self-referential thinking, autobiographical memory retrieval, imagining the future, and mentalizing. In contrast, the task-positive network becomes active during externally focused, goal-directed activities that require attention, cognitive control, and problem solving. These two networks have an antagonistic relationship: when task demands increase, activity in the task-positive network rises while activity in the DMN is suppressed, and when attention subsides, the DMN re-emerges, allowing the brain to flexibly alternate between internal reflection and engagement with the external environment (Uddin et al., 2009).

In people with ADHD, the DMN appears to deactivate less efficiently during tasks requiring attention. Specifically, certain DMN regions exhibit weaker internal connectivity and reduced coupling with frontal brain regions that normally regulate its activity and coordinate the activation of the task-positive network (Castellanos et al., 2008). This atypical DMN connectivity impairs suppression of the DMN during cognitively demanding tasks, allowing internally generated thoughts to intrude (Sonuga-Barke & Castellanos, 2007). As a result, the DMN remains active in the background, contributing to mind-wandering, distractibility, and difficulties sustaining attention. Such altered network dynamics may help explain the commonly reported experience in ADHD of a persistent conflict between the intention to focus and the pull of task-unrelated thoughts (Sripada et al., 2014).

Relevant insights into this phenomenon are provided by Danckert and Merrifield (2018), who examined neural activity in a small group of healthy adults (*N* = 13) across four experimental conditions: an interest induction (viewing a documentary nature film), a boredom induction (repeated viewing of a monotonous clip depicting two men hanging laundry), a sustained attention task (detecting the appearance of new stars in a static night sky), and a resting-state condition (passive fixation on a cross). Participants reported significantly higher levels of state boredom during the boredom, sustained attention, and resting-state conditions relative to the interest induction condition. Notably, these boredom-associated conditions were also characterized by increased correlated activation in posterior regions of the DMN.

Taken together, these findings suggest a potential mechanistic link between boredom and DMN engagement. In healthy individuals, boredom appears to coincide with a shift towards DMN-dominated processing, reflecting disengagement from externally focused tasks and a turn toward internally generated cognition. In the context of ADHD—where DMN suppression is already compromised—this boredom-related DMN activation may be especially pronounced. From the perspective of the cognitive-energetic model of ADHD (Sergeant, 2000), such low-stimulation or monotonous task contexts are associated with suboptimal arousal and activation states, reducing the energetic support available for sustained task engagement. As a result, individuals with ADHD may be particularly vulnerable under these conditions, as diminished energetic regulation may further exacerbate DMN intrusions and undermine attentional control. This greater impairment in DMN suppression may help explain why individuals with ADHD often experience boredom as a cognitively disruptive state characterized by intensified mind-wandering that competes with task-relevant processing (Bozhilova et al., 2018).

## Executive Dysfunction and Attention Impairment

Executive functions are higher-order cognitive processes that allow individuals to regulate behavior, thoughts, and emotions in a goal-directed and adaptive or functional manner. Core executive functions—such as working memory, inhibition, and cognitive flexibility—support complex skills including planning, reasoning, problem-solving, and decision-making (Diamond, 2013). These processes are primarily linked to frontal brain networks and are crucial for managing novel or demanding situations where automatic responses are insufficient. By coordinating lower-level cognitive processes (such as perception, memory, and automatic responses), executive functions help individuals organize information, monitor performance, and adjust strategies to achieve desired outcomes (Hofmann et al., 2012).

Attention is a fundamental component of executive functions, providing the ability to allocate, sustain, and flexibly shift cognitive resources in support of goal-directed behavior (Petersen & Posner, 2012). At the same time, executive functions influence how attention is deployed, helping to prioritize relevant information, suppress distractions, and maintain focus on goals (Kaplan & Berman, 2010). For instance, inhibitory control relies on attention to resist irrelevant information, while cognitive flexibility allows for strategic shifting of attention between tasks or mental representations. In this way, attention and executive functions operate in a dynamic, reciprocal system, each shaping and supporting the other to enable purposeful, controlled behavior.

Barkley’s (1997) widely cited model of ADHD proposes that impairments in executive functions and attention lie at the core of the disorder, contributing to many of its behavioral and cognitive difficulties. A meta-analysis by Wilcutt et al. (2005) synthesized 83 studies including individuals with ADHD (*n* = 3734) and controls (*n* = 2969) to evaluate the empirical support on this notion. The analysis found that those with ADHD exhibited significant impairments across a range of executive functioning and attention tasks, with effect sizes ranging between *d* = .46–.69). Translating these effect sizes into a binomial effect size display suggests that roughly 61–66% of individuals with ADHD show marked deficits in executive functions, an effect that can be considered moderate. This indicates that—although not universal—executive function and attention impairments are common in ADHD.

Interestingly, Eastwood et al. (2012) conceptualize boredom as a state closely tied to deficits in attention and executive function. They argue attentional control and executive processes—such as planning, initiation, and self-regulation—play a critical role in generating and sustaining engagement, particularly in situations that are low in external stimulation. When these functions are intact, individuals can actively direct attention and create meaningful internal or external activity despite limited environmental input. However, when attention and executive functions are compromised, as is often the case in ADHD, individuals may struggle to initiate or maintain such engagement. This difficulty in internally or externally involving oneself in meaningful activity can leave situations feeling uninteresting or effortful, thereby increasing the likelihood of experiencing boredom.

## Altered Reward Sensitivity

Reward can play a central role in learning and motivation by reinforcing behaviors that lead to positive outcomes, thereby increasing the likelihood that those behaviors will be repeated. Experience such as praise, achievement, or tangible incentives activate the brain’s reward system, strengthening associations between actions and their consequences and supporting skill acquisition and goal-directed behavior (Berridge & Robinson, 2003). Individuals differ, however, in their sensitivity to reward, which shapes how effectively incentives motivate behavior. Those with higher reward sensitivity tend to respond more strongly to positive feedback and incentives, thereby showing greater engagement and persistence when rewards are salient. Rewards may be extrinsic, such as money, grades, or social approval, or intrinsic, arising from interest, enjoyment, or a sense of mastery. While extrinsic rewards can effectively initiate and sustain behavior, intrinsic motivation is more closely linked to deeper learning, creativity, and long-term engagement, as behavior is driven by inherent interest rather than external reinforcement (Deci & Ryan, 2000).

Evidence suggests that individuals with ADHD show systematic differences in reward processing and reward sensitivity compared to neurotypical individuals (Luman et al., 2010, 2012). In ADHD, motivation is more strongly influenced by immediacy: rewards that are immediate, frequent, and salient exert a greater motivating effect, whereas delayed or inconsistent gratifications rapidly lose their rewarding impact. These patterns are thought to reflect altered functioning in dopamine-mediated brain circuits involved in reinforcement learning, motivation, and reward anticipation (Volkow et al., 2011). Consequently, individuals with ADHD may demonstrate reduced anticipation of future rewards, weaker learning from delayed feedback, and heightened responsiveness to novel or high-intensity rewards (Sonuga-Barke, 2005). Such differences help explain characteristic behavioral patterns in ADHD, including difficulty sustaining effort on long-term tasks, a preference for activities with quick payoff, and marked fluctuations in motivation depending on how and when rewards are delivered.

These alterations in reward processing are closely related to heightened experiences of boredom in ADHD. When tasks or contexts provide limited stimulation, delayed feedback, or low-saliency rewards, they may fail to sufficiently engage the reward system, leading to a rapid decline in motivation. This diminished reward signaling can result in disengagement and a subjective experience of boredom (Danckert & Eastwood, 2020), even in contexts that others may find manageable or mildly engaging. Thus, boredom in ADHD appears to reflect an underlying mismatch between reward system functioning and environments that provide sparse, delayed, or predictable reinforcement, resulting in a heightened need for novelty, intensity, or immediate payoff to maintain engagement.

## Lack of Meaning

A sense of meaning or purpose is a core component of healthy psychological functioning. Meaning allows individuals to organize their experiences, establish goals, and endure adversity by providing a framework through which life events are understood as significant. In this way, meaning supports motivation and self-regulation, guiding behavior over time and enabling persistence in the face of stress and suffering (Baumeister, 1991). Empirical research consistently demonstrates that individuals who perceive their lives as meaningful report higher levels of well-being and life satisfaction, as well as lower levels of depression and anxiety than people who experience their lives as less meaningfully (He et al., 2023; Li et al., 2021).

Individuals with ADHD often encounter specific challenges that can complicate the development and maintenance of a coherent sense of meaning or purpose. Difficulties with long-term planning may render goals abstract, unstable, or easily disrupted, reflecting core executive function deficits in planning and self-regulation associated with ADHD (Barkley, 1997). In addition, impairments in time perception—often described as “time blindness”—can hinder the ability to link present and past actions to future outcomes, undermining sustained engagement in purpose-driven behavior (Weissenberger et al., 2021). Furthermore, inconsistent performance, chronic underachievement relative to ability, and ongoing difficulties with self-regulation can contribute to repeated experiences of unmet expectations and external criticism (Jensen et al., 2025). Over time, these experiences are associated with reduced self-esteem and disruptions in self-concept among individuals with ADHD (Cook et al., 2014).

Contemporary accounts of boredom emphasize the role of meaning in the experience of boredom (Danckert & Eastwood, 2018; Westgate & Wilson, 2018). Specifically, boredom is thought to arise via two primary routes. The first route concerns situational contexts in which individuals perceive a discrepancy between their current activity and the goals they seek to achieve. For instance, Van Tilburg and Igou (2012) demonstrated experimentally that participants instructed to engage in purposeless activities (e.g., copying bibliometric entries on concrete mixtures) experienced these tasks as meaningless and subsequently reported heightened boredom. The second route pertains to the individual differences in *meaning in life*, defined as the general sense that one’s existence is significant, purposeful, and coherent. From this perspective, individuals with a diminished sense of life meaning are expected to experience boredom more frequently than individuals with a heightened sense of life. Supporting this view, Fahlman et al. (2009) assessed meaning in life and boredom at two time points and found consistent negative associations between the two constructs. Importantly, their longitudinal analyses suggested lower meaning in life may precede increases in boredom over time. Both routes seem relevant for people with ADHD, who often encounter tasks that—due to characteristic difficulties with inattention and hyperactivity/impulsivity—are challenging to complete (route 1). Repeated experiences of task failure and downstream functional consequences (French et al., 2024) may, over time, undermine their sense of existential meaningfulness.

## ADHD and Boredom: An Integrative Framework

The ADHD-boredom link is relevant throughout the entire lifespan, but in Western and other industrialized countries it is particularly pronounced during adolescence and young adulthood, when individuals face the most burdensome phase of the education system (Lewczuk et al., 2024). This period is often framed as laying the foundation for a prosperous future and, in many countries, culminates in the highest attainable level of formal education. In most cases, this system poses substantial challenges for individuals with ADHD (De Zeeuw et al., 2017), as schools and universities traditionally rely on one-way instructional methods combined with extensive self-study (e.g., homework assignments), with progress primarily assessed during examination periods. In such environments, which offer limited stimulation, autonomy, and immediate feedback, boredom is ever-present (Tze et al., 2016)—especially for individuals with ADHD, whose neurocognitive profile is characterized by suboptimal regulation of attention, arousal, and reward processing, making sustained engagement in monotonous, externally paced tasks particularly difficult. This is illustrated in Box 1, which presents a case vignette of an adolescent with ADHD experiencing chronic boredom in the educational context.

**Fig. 3:**
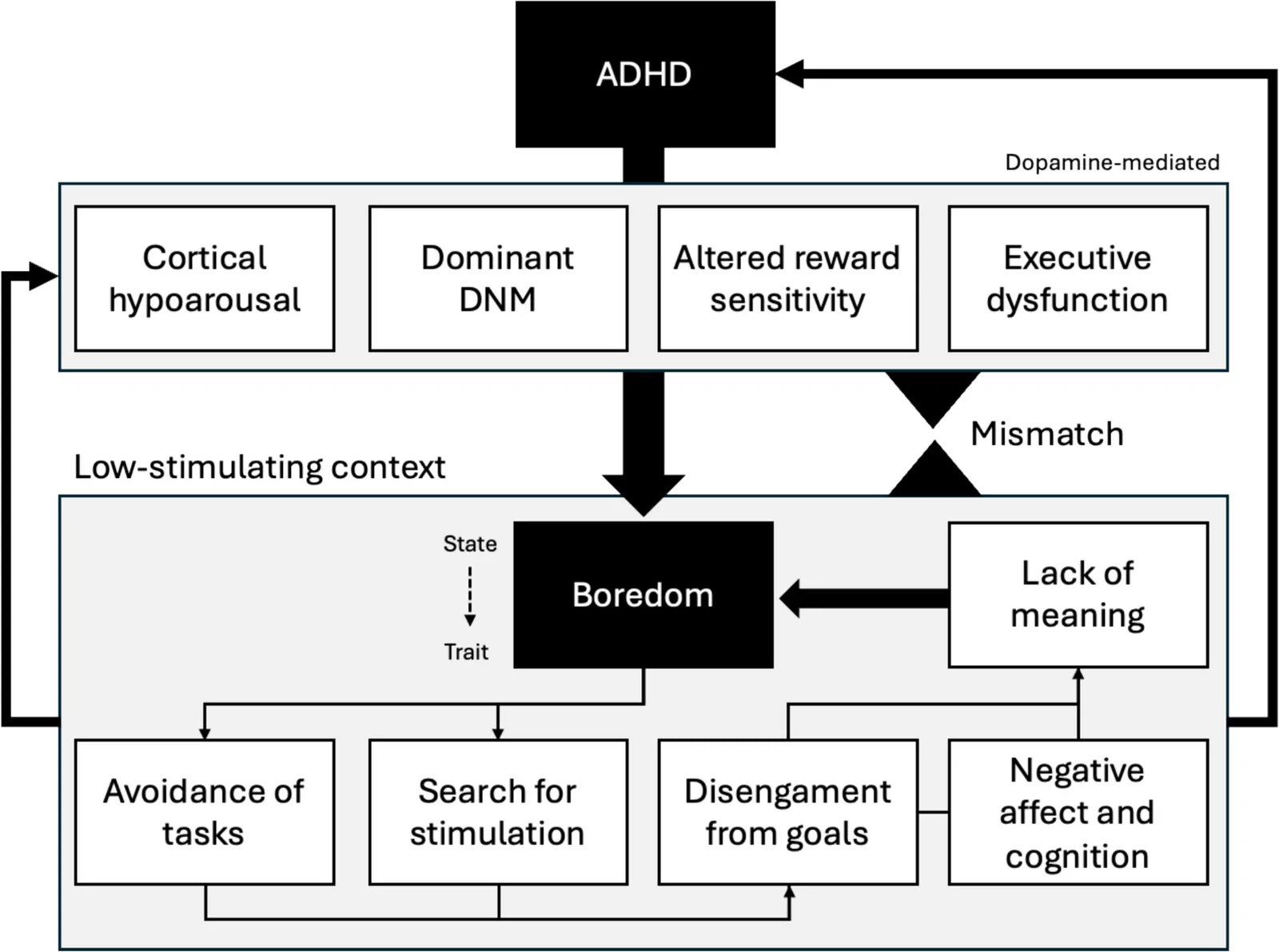
An integrative model linking ADHD-related neurocognitive characteristics to boredom and its downstream behavioral and emotional consequences. *Note*. ADHD = Attention-Deficit/Hyperactivity Disorder, DNM = Default Network Mode

Previous sections have summarized theoretical accounts relevant to understanding the nexus between ADHD and boredom. Figure 3 presents a conceptual model that integrates these perspectives. At the top of the model, ADHD is characterized as a neurodevelopmental disorder involving multiple aberrant brain-related mechanisms, such as cortical hypoarousal, dominant DMN activity, altered reward sensitivity, and executive dysfunction—including attentional dysregulation. These mechanisms are largely dopamine-mediated and are likely intercorrelated, yet each contributes logically to the prototypical ADHD phenotype, which is characterized by distractibility, behavioral restlessness, and impulsivity.

When individuals with ADHD encounter low-stimulating contexts in which a task has to be accomplished, a pronounced mismatch emerges: their neural systems fail to sufficiently “switch on”, making it difficult to mobilize the cognitive resources necessary for task completion. This under-engagement leads to an appraisal of the situation as unstimulating, which in turn elicits boredom. Once boredom arises, multiple pathways unfold. It is associated with task avoidance and an increased drive to seek stimulation (Bieleke et al., 2022), which can ultimately result in disengagement from goals and heightened negative affect (Danckert & Eastwood, 2020). Over time, this can generate a perceived lack of meaning, which feeds back into the experience of boredom, forming a reinforcing loop. When this cycle occurs repeatedly, boredom may become a dominant and habitual emotional response in low-stimulating situations.

Importantly, boredom-related emotional and behavioral responses not only emerge from ADHD-related mechanisms but also contribute to their maintenance and amplification. Task avoidance in response to boredom mirrors and reinforces inattentive symptoms by limiting sustained engagement and persistence (Diamond, 2005). Simultaneously, boredom-driven attempts to upregulate arousal—through motor activity, novelty seeking, or rapid task switching—manifest behaviorally as hyperactivity and impulsivity (Kiliç et al., 2020; Matthies et al., 2012). Beyond sustaining the behavioral phenotype, the emotional and motivational consequences of boredom can further strain underlying brain systems implicated in ADHD. Boredom-related negative affect, including frustration and irritability, increases demands on already compromised executive control systems, thereby further impairing working memory, inhibitory control, and sustained attention (Pessoa, 2009). In parallel, repeated reliance on highly salient, immediately rewarding activities—such as digital media use or gaming (Tagliaferri et al., 2025) or substance use (Iso-Ahola & Crowley, 1991; Weybright et al., 2015)—may bias reward-processing systems toward immediate reinforcement and reduce tolerance for effort and delay (Raiha et al., 2020). This pattern exacerbates existing dopaminergic alterations, decreases the subjective value of low-stimulation tasks, and widens the mismatch between environmental demands and neural engagement.

Overall, this integrative model illustrates how ADHD-related neural and cognitive mechanisms give rise to situational boredom, which can evolve into a habitual propensity for boredom. In turn, boredom drives maladaptive motivational, emotional, and behavioral outcomes through self-perpetuating cycles. Task avoidance reinforces inattentive behavior, while stimulation-seeking responses manifest as hyperactivity and impulsivity, sustaining the core behavioral phenotype. Concurrently, the emotional and cognitive strain associated with boredom further challenges executive control systems, intensifies negative affect, and biases reward processing toward immediate gratification. By linking neural mechanisms, cognitive deficits, and behavioral tendencies, the model highlights how situational boredom is both a consequence of ADHD and a factor that reinforces and amplifies its core symptoms. Understanding these feedback loops provides a framework for conceptualizing why individuals with ADHD may be particularly prone to boredom and the downstream effects this has on motivation, emotion regulation, and goal-directed behavior.

Box 1 Case vignette illustrating the role of boredom in ADHD *“Tom”* is a 15-year-old boy with a diagnosis of ADHD who illustrates how high cognitive potential can coexist with substantial educational difficulties, particularly in the context of chronic boredom. Cognitive assessment revealed above-average to superior intellectual functioning, with verbal and non-verbal IQ scores ranging from 118 to 130, alongside relatively weaker working memory and processing speed. During primary school, Tom was treated with stimulant medication, which supported his academic functioning. Upon entering secondary school, however, he decided to discontinue medication, motivated by a desire to cope independently.

Tom initially enrolled in a pre-university educational track but struggled to adapt to the instructional demands of the system. Classroom teaching was largely experienced as slow-paced and repetitive, and he frequently reported feeling bored and mentally under-stimulated. Sustaining attention during lessons proved difficult, as did planning, organizing, and completing daily homework assignments. These difficulties intensified during examination periods, when large amounts of information had to be processed through prolonged self-study—an educational format he experienced as particularly tedious and disengaging.

Although Tom began secondary school with high motivation, his engagement gradually declined. As tasks increasingly felt meaningless and unrewarding, his effort declined, and his academic performance steadily worsened. This resulted in a setback to a lower educational level. Concurrently, Tom experienced growing anger and frustration, loss of confidence, and a marked decrease in motivation to invest in what he described as “boring schoolwork.” School absenteeism increased, and to cope with his negative feelings and academic stress, he began using cannabis with peers*, while online gaming became a dominant activity in his daily routine.

Despite these challenges, Tom took an additional year to complete secondary school and ultimately obtained his diploma. However, he was unable to initiate further education. Instead, he entered low-paid employment, where he functioned well in a more structured, active, and immediately rewarding environment. He was quickly entrusted with greater responsibilities, as his supervisor noted that Tom appeared considerably more intellectually capable than most of his coworkers.

^*^Cannabis use is frequently reported among individuals with ADHD as a strategy to cope with boredom and negative affect; however, evidence indicates that cannabis has detrimental effects on cognition that may ultimately exacerbate these difficulties (Hernandez & Levin, 2024)

## Implications for Intervention

Building on this integrative model, one clear implication is that pharmacological interventions commonly used in ADHD may also attenuate boredom by targeting the neurocognitive mechanisms that give rise to it. Stimulant medications that enhance dopaminergic signaling (Jaeschke et al., 2021; Volkow et al., 2002) are likely to reduce boredom experiences by facilitating engagement of the task-positive network while suppressing DMN activity (Santos et al., 2019), improving attentional stability and executive control (Tamminga et al., 2016), increasing cortical arousal (Rubia et al., 2014), and optimizing reward sensitivity (Furukawa et al., 2020). Together, these effects may render goal-directed tasks more intrinsically engaging and reduce the subjective under-stimulation that characterizes boredom in ADHD. Within the framework of the model, medication-related improvements in attention, reward processing, and cognitive control would therefore be expected to disrupt the self-perpetuating cycles linking boredom to task avoidance, negative affect, and impulsive stimulation seeking. Empirical findings to date are consistent with this interpretation, suggesting that reductions in ADHD symptom severity during stimulant treatment are accompanied by parallel reductions in boredom proneness (Golubchik et al., 2020). However, the existing evidence base remains limited, and further longitudinal and experimental studies are needed to more directly examine medication-mediated effects on boredom, clarify underlying neural mechanisms, and determine whether reductions in boredom contribute independently to functional improvements beyond core symptom change.

Another promising avenue for intervention involves reducing the mismatch between the neurodivergent brain of individuals with ADHD and the low-challenging and -stimulating demands typical of many school and workplace settings. Research shows that features of the environment—such as classroom size and spatial configuration—significantly influence attention and task engagement in people with ADHD, with smaller class sizes and more spacious contexts linked to better attentional performance (Khozaei et al., 2025). Population-level interventions that increase structure, predictability, and positive reinforcement (e.g., School-Wide Positive Behavioral Interventions and Supports) are associated with reduced need for pharmacotherapy and improved adjustment, suggesting that environmental redesign can attenuate ADHD-related difficulties (Borgen et al., 2021). Complementing these structural interventions, individuals with ADHD frequently report that highly stimulating, novel, and intrinsically interesting work environments help them focus more effectively and experience their strengths rather than impairments, indicating that context can modulate symptom expression and functional outcomes (Lasky et al., 2016). School climate factors such as academic engagement, disciplinary structure, and supportive relationships have also been longitudinally linked with better adaptive functioning in adolescents with ADHD (Chan et al., 2025). These findings align with ecological models of ADHD that emphasize person-environment fit (Nguyen et al., 2019) and underscore the potential for context modifications—such as tailored tasks, enriched stimuli, flexible pacing, reinforcement, or accommodations—to reduce boredom, bolster engagement, and lessen downstream cognitive-affective and behavioral sequelae. However, direct tests of whether environmental changes reduce boredom specifically are largely lacking. Likewise, it remains unclear whether reductions in boredom mediate improvements in motivation and self-regulation, highlighting a critical direction for future research.

Psychoeducation is an important component of the clinical treatment of individuals with ADHD and has been shown to yield positive outcomes, including improvements in clinical symptoms, social skills, general functioning, and interpersonal relationships (Syed et al., 2024). Within psychoeducation packages, emotions and emotional dysregulation are typically addressed (Morris et al., 2025a, 2025b); however, the focus is largely limited to anger or frustration, sadness, and anxiety (Beheshti et al., 2020). Boredom, despite its presumed relevance to ADHD, is often neglected and could represent a valuable addition to these interventions. Including boredom in psychoeducation may help individuals reframe it as a meaningful emotional and motivational signal rather than a personal shortcoming, thereby reducing feelings of shame and self-blame. Furthermore, teaching strategies to manage boredom—such as modifying tasks, enhancing intrinsic or extrinsic rewards, and pacing cognitive effort—may improve engagement, persistence, and self-regulation in everyday contexts. Explicitly addressing boredom may therefore enhance the ecological validity of psychoeducation packages and promote a comprehensive understanding of emotional experiences and functional coping in individuals with ADHD.

A final intervention venue involves targeting boredom and its associated behavioral and cognitive sequelae through psychological interventions such as acceptance and commitment therapy (ACT; Hayes, 2004). ACT may be particularly well suited for individuals with ADHD, as it emphasizes acceptance of aversive internal experiences—such as boredom, restlessness, and low stimulation—rather than efforts to avoid or eliminate them, which inadvertently intensify disengagement and impulsive behavior (Munawar et al., 2021). By cultivating psychological flexibility, ACT supports sustained engagement in valued activities even when tasks are experienced as unstimulating, a core difficulty in ADHD. Moreover, ACT’s emphasis on cognitive defusion may attenuate the impact of boredom-related negative self-judgments (e.g., perceiving oneself as lazy or unmotivated), while its focus on values-based action promotes persistence and goal-directed behavior despite fluctuating motivation. Importantly, ACT interventions may also enhance individuals’ sense of purpose and meaning in life, thereby providing a broader motivational framework that can buffer against chronic boredom and disengagement. Taken together, ACT offers a promising therapeutic framework for addressing boredom as a central emotional and motivational challenge in ADHD.

## Conclusion

While the relationship between ADHD and boredom may seem obvious, this has paradoxically led to the phenomenon being understudied. Our literature search yielded only 18 studies, of which a limited number (*k* = 8, 44%) explicitly examined the link between ADHD and boredom. Moreover, the existing research is far from optimal. Most studies focus on adults rather than on children and adolescents, despite the childhood onset of ADHD. Samples are often skewed toward females, which contrasts with the known male–female ratio in ADHD diagnoses, and studies are more frequently conducted in non-clinical populations than in clinical ones. Notably, those studies that do rely on clinical samples are predominantly focused on youths, leaving clinically referred adults with ADHD largely understudied. In addition, boredom has primarily been assessed through self-report measures, limiting the validity and generalizability of the conclusions that can be drawn.

This modest scientific interest may reflect the widespread assumption that boredom is merely an aspecific by-product of ADHD, secondary to core symptoms such as inattention or impulsivity. In contrast, we argue that boredom represents a more central and influential emotional experience in ADHD. Rather than being epiphenomenal, boredom may play a key role in linking aberrant brain functioning to negative behavioral, emotional, cognitive, and experiential consequences that shape long-term outcomes in individuals with ADHD.

To further substantiate this perspective, future research should explicitly examine the relationship between ADHD and boredom using more representative samples, including clinical populations, children and adolescents, more balanced male–female ratios, and multi-informant approaches. Prospective survey studies could help determine whether boredom predicts negative outcomes in individuals with ADHD beyond the effects of symptom severity alone. Experimental studies that systematically induce boredom would allow researchers to explore ADHD-related cognitive, behavioral, emotional, and experiential responses to this state (cf. Havermans et al., 2015). In addition, future work could explore the relationship between boredom and Cognitive Disengagement Syndrome (CDS; previously termed sluggish cognitive tempo; Becker, 2025; Frederick & Becker, 2023), which has been shown to be associated with mind-wandering above and beyond ADHD symptoms (Frederick et al., 2020). Examining whether the boredom-CDS association is comparable to that observed for ADHD would help clarify the specificity of boredom-related processes across attentional phenotypes. Finally, intervention research is needed to investigate whether pharmacological treatments—replicating and extending findings such as those of Golubchik et al. (2020)—as well as psychological approaches, including psychoeducation, improving person–environment fit, and ACT, can effectively reduce boredom and, in turn, alleviate ADHD-related difficulties. Advancing research in these directions will help clarify the role of boredom as a meaningful target for both theory and treatment in ADHD.

## Data Availability

No datasets were generated or analysed during the current study.
